# CO_2_ Conversion into N-Doped Porous Carbon-Encapsulated NiO/Ni Composite Nanomaterials as Outstanding Anode Material of Li Battery

**DOI:** 10.3390/nano10081502

**Published:** 2020-07-31

**Authors:** Yayong Li, Chunxiao Xu, Kaiyuan Liu, Pengwan Chen, Xin Gao

**Affiliations:** State Key Laboratory of Explosion Science and Technology, Beijing Institute of Technology, No. 5 Zhongguancun South Street, Haidian, Beijing 100081, China; 2120170226@bit.edu.cn (Y.L.); 3120140441@bit.edu.cn (C.X.); 13121164743@163.com (K.L.)

**Keywords:** combustion synthesis, CO_2_ reduction, porous carbon, nickel oxide, hydrazine hydrate, lithium ion battery, nanocomposite

## Abstract

N-doped porous carbon encapsulated NiO/Ni composite nanomaterials (N-doped NiO/Ni@C) was successfully obtained by a one-step solution combustion method. This study demonstrates a one-step combustion method to synthesize n-doped porous carbon encapsulated NiO/Ni composite nanomaterials, using carbon dioxide as the carbon source, nickel nitrate as the nickel source, and hydrazine hydrate as the reaction solution. Spherical NiO nanoparticles with a particle size of 20 nm were uniformly distributed in the carbon matrix. The load of NiO/Ni can be controlled by the amount of nickel nitrate. The range of carbon content of recovered samples is 69–87 at%. The content of incorporated nitrogen for recovered samples is 1.94 at%. As the anode of lithium ion battery, the composite material exhibits high capacity, excellent multiplier performance and stable circulation performance. N-doped NiO/Ni@C-2 was applied to lithium ion batteries, and its reversible capacity maximum is 980 mAh g^−1^ after 100 cycles at the current density of 0.1 A g^−1^. Its excellent electrochemical properties imply its high potential application for high-performance lithium-ion battery anode materials.

## 1. Introduction

The lithium-ion battery (LIB) is the most advanced high-efficiency secondary battery and the fastest-developing chemical energy storage battery. Owing to the LIB’s high specific energy, low self-discharge, superior cycle performance, no memory effect, and environmental friendliness, it remains a subject of great interest [1]. The high electrochemical performance of lithium ion batteries is affected critically by the anode material. At present, the commercial anode material is graphite limited in practical applications due to its low theoretical specific capacity, which does not match the development requirements of mobile electronic equipment [2]. Therefore, new anode materials have been a hot topic of research. Poizot et al. [3] reported that nano-sized transition metal oxides exhibit good electrochemical properties as anode materials for lithium ion batteries, such as Co_3_O_4_ [4], NiO [5], MnO_2_ [6], CuO [7], V_2_O_5_ [8], etc.

Among them, NiO is an ideal anode material owing to its high lithium storage capacity (718 mAh g^−1^) [9] and low cost. However, the volume effect caused by the decomposition of NiO decrease the capacity of Li battery rapidly and further reducing the cycle stability [10]. Various NiO nanostructures, such as nanoparticles, nanosheets, nanowires, etc. [11,12,13], have been applied in the anode of lithium ion batteries to increase the specific surface area of NiO and further shorten the path of lithium ion diffusion. Liu et al. [14] employ a facile bio-template engaged route using filter paper as the template to prepare NiO hollow nanotubes exhibiting a capacity of 600 mAh g^−1^ after 100 cycles at 200 mAh g^−1^ current density. Furthermore, several carbon-based NiO nanocomposite materials were synthesized to increase the conductivity and specific surface area of NiO material for a higher performance on the cycle stability of the LIB. Wu et al. [15] synthesized three dimensional (3D) Ni foam/N-CNTs/NiO nanosheets as an electrode material by both chemical vapor deposition and electrochemical deposition methods. This electrode material exhibits a larger capacity, better cycling stability, superior rate capability, and higher ionic conductivity. Li et al. [16] obtained one-dimensional (1D) porous TiO_2_-carbon nanofibers (ODPTCNs) by a simple coaxial electrospinning technique combined with subsequent calcination treatment. The novel ODPTCNs as an anode material for LIB show a remarkable specific reversible capacity of 806 mAh g^−1^ and a high volumetric capacity of 1.2 Ah cm^−3^ and exhibit an exceptional discharge rate capability of 5 A g^−1^ while retaining a capacity of 260 mAh g^−1^ after 1600 cycles. Zhao et al. [17] synthesized a unique two-dimensional (2D) graphene/NiO composite material by self-assembly of Ni ions directly on the surface of graphene oxide. The synthesized material exhibits an excellent surface area (134.5 m^2^ g^−1^) and electrochemical performance.

Note that many other approaches can be utilized to further increase the electrochemical performance of materials, such as doping technique, increase of specific area, and synthesis of composite materials. Nitrogen doped carbon materials exhibit higher capacitance in addition to fast charge/discharge characteristics owing to the pseudo-capacitive effect of incorporated nitrogen [18]. Moreover, the higher active reaction site on these materials is conducive to the enhancement of electrochemical performance [19]. In addition, porous carbon nanomaterials possess high porosity, high specific surface area, and good electron mobility [20], which can be also utilized as matrix materials to enhance the electrochemical performance of LIB anode material. Moreover, metal/metal oxide composite material features high electrical conductivity, rapid transmission of electrons during the reaction and good specific surface area. Lai et al. [21] reported the novel mesostructured NiO/Ni composites, which consist of hetero-NiO/Ni components at the nanoscale while displaying 3D porous architectures at the mesoscale, with an adjustable metallic Ni content in a wide range. The NiO/Ni composites exhibit high structural stability and high circulation times owing to its abundant reaction sites and rapid progress for the redox reaction, and high rate capability and specific capacity.

Solution combustion synthesis (SCS), also known as low temperature combustion synthesis (LCS), is a new route to prepare materials, featuring low reaction temperature [22,23,24]. In the SCS method, a liquid mixture of reductant and oxidant is heated to trigger the self-propagating combustion with an intense self-exothermic process. Through the reaction, nanopowders were obtained with generation of a large amount of gas.

In our previous work, nitrogen doped carbon nanomesh sheets were successfully synthesized by SCS in ethanolamine medium [25]. The obtained materials exhibit high specific surface area of 888 m^2^ g^−1^, high pore volume of 2.05 cm^3^ g^−1^, good pore structure, and high content of 2.88 at% nitrogen in situ doping, and exhibit an excellent electrochemical performance in fuel cell. However, the synthesis of carbon-based nanocomposite materials and corresponding electrochemical properties have been rarely reported.

In this work, we employ carbon dioxide as carbon source, nickel nitrate as the precursor of nickel oxide and nickel, and hydrazine hydrate as adsorption solution to synthesize N-doped NiO/Ni@C composite nanomaterials via SCS method. The synthesized N-doped NiO/Ni@C materials exhibit the specific surface area of 326 m^2^ g^−1^, the pore volume of 1.23 cm^3^ g^−1^, and high content incorporated nitrogen of 1.94 at%. The above properties indicate that N-doped NiO/Ni@C is an ideal anode material for LIB.

## 2. Experimental

### 2.1. Synthesis of NiO/Ni@C

**Figure 1:**
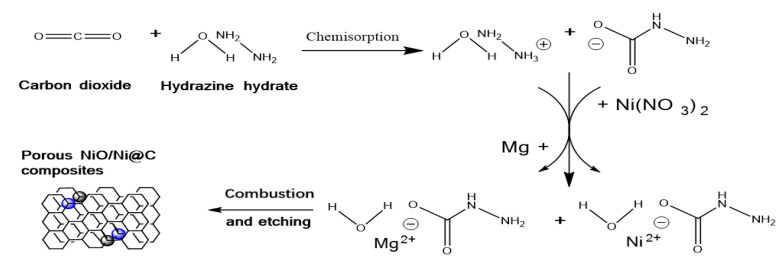
The principle of combustion synthesis of N-doped NiO/Ni@C.

The experimental flow chart is shown in Figure 1. Alkaline hydrazine hydrate solution (Shanghai Aladdin Bio-Chem Technology Co., Ltd., Shanghai, China) was selected as reaction solution. Carbon dioxide gas (Beijing Tianli Renhe Materials Trading Co., Ltd., Beijing, China) was adsorbed and hydrolyzed into the reaction solution at room temperature to form hydrazine formic acid (N_2_H_4_COOH) (see Equation (1)). Then, three hydrazine precursor solution specimens (10 g) were poured into three quartz crucibles, respectively. Subsequently, three nickel nitrate powders with different mass (0.5 g, 1 g, and 2 g, Beijing Tongguang Fine Chemical Company, Beijing, China) were added into three solution specimens to spontaneously complexate with hydrazine formic acid (Equation (2)). After that, excess magnesium powder (2.5 g, Beijing Tongguang Fine Chemical Company, Beijing, China) was added into each solution specimen and spontaneously reacted with ammonium cation hydrolyzed to form magnesium ion which complexated with hydrazine formic acid to form a homogeneous sol precursor ((NH_2_NHCOO)_2_Mg and (NH_2_NHCOO)_2_Ni). During the above process, the released gas (H_2_, see Equation (3)) leads to the mixture of the residual magnesium powder after a previous reaction and liquid solution. Then, the self-propagating combustion of residual Mg powder and homogeneous sol precursor is ignited by tungsten filament. The synthesized grey powders were recovered and etched by 1M HCL (Beijing Tongguang Fine Chemical Company, Beijing, China) for 30 min to remove MgO impurities, and further filtrated. The final products were dried and labeled as NiO/Ni@C-0.5, NiO/Ni@C-1, NiO/Ni@C-2.

2NH_2_NH_2_·H_2_O + CO_2_→NH_2_NHCOO^−^ + NH_2_NH_3_^+^ + 2H_2_O, (1)

2NH_2_NH_3_^+^ + 2NH_2_NHCOO^−^ + Ni^2+^→(NH_2_NHCOO)_2_Ni + 2NH_2_NH_2_, (2)

2NH_2_NH_3_^+^ + 2NH_2_NHCOO^−^ + Mg→(NH_2_NHCOO)_2_Mg + 2NH_2_NH_2_ + H_2_, (3)

### 2.2. Characterizations

The morphology of the samples was characterized by ZEISS Sigma-500 scanning electron microscope (SEM, Carl Zeiss AG, Jena, Germany) and JEM-2100Plus transmission electron microscope (TEM, JEOL, Tokyo, Japan). Bruker D8 Advance diffractometer was used to test the X-ray diffraction (XRD, Bruker (Beijing) Scientific Technology Co., Ltd., Beijing, China). Nitrogen adsorption and desorption experiments were carried out with Quantachrome Autosorb-IQ-MP device (Anton-Paar China, Shanghai, China) under the condition of 77 K. Before nitrogen adsorption, the sample was degassing under vacuum at 243 K temperature for 10 h. Specific surface area (SSA) and pore diameter distribution were obtained by Brunauer Emmett Teller (BET) and density functional theory (DFT). X-ray photoelectron spectroscopy (XPS) testing was carried out on ESCALab 250 (Thermo Fisher Scientific, Walsham, MA, USA). Raman spectra were measured using the French LabRAM Aramis microconfocal Raman spectrometer (Horiba Jobin Yvon, laser with a wavelength of 532 nm, Paris, France).

### 2.3. Anode Preparation of Lithium Ion Battery

The synthesized samples were mixed with conductive additives (Timcal Super C65, Shanghai Aladdin Bio-Chem Technology Co., Ltd., Shanghai, China) and polyvinylidene fluoride (PVDF, Shanghai Aladdin Bio-Chem Technology Co., Ltd., Shanghai, China) bindered in 1-methyl-2-pyrrolidinone (NMP, Shanghai Aladdin Bio-Chem Technology Co., Ltd., Shanghai, China) along a mass ratio of 8:1:1, and then coated on the copper foil collector and dried under 80 °C for 12 h in vacuum to prepare working electrode with a diameter of 12 mm and a mass of approximately 2.5 mg cm^−2^. Electrochemical testing and cycling were conducted in a button-type 2032 half battery, with lithium metal as the counter electrode. The separator was Celgard 2500 polypropylene with 1M LiPF_6_ electrolyte dissolved in a mixture of ethylene carbonate (EC, Shanghai Aladdin Bio-Chem Technology Co., Ltd., Shanghai, China) and dimethyl carbonate (DMC, Beijing Tongguang Fine Chemical Company, Beijing, China) (V_EC_:V_DMC_ = 1:1). In an Arbin circulation system with the same current density of 0.1 A g^−1^, the regulated voltage is 0.005–3 V (vs. Li^+^/Li) at room temperature.

## 3. Results and Discussion

### 3.1. Characterization Results of Recovered Samples

**Figure 2:**
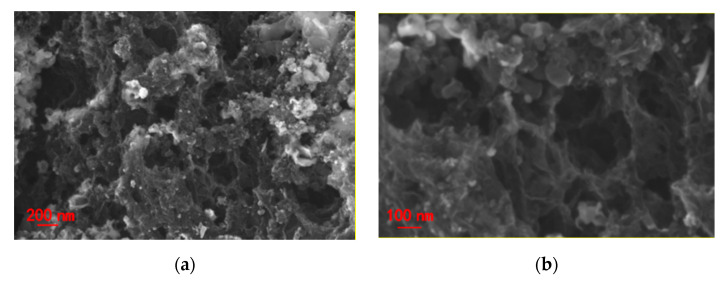

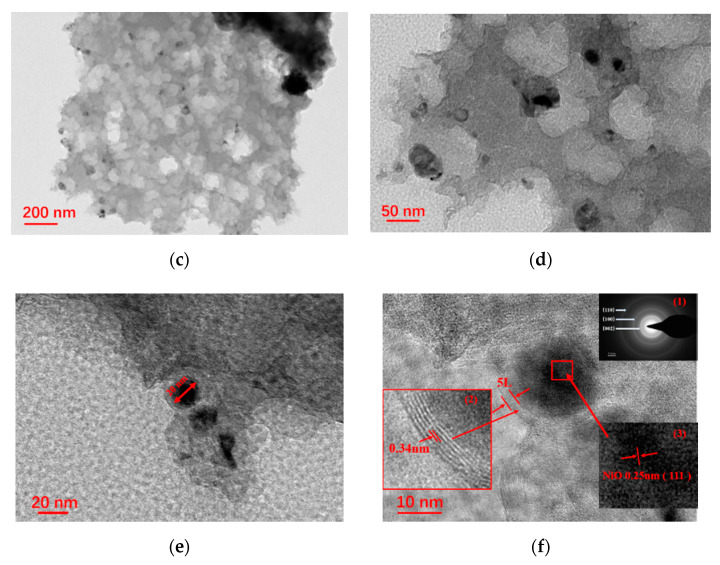
NiO/Ni@C-2 composite nanomaterials (**a**,**b**) SEM (scanning electron microscope), (**c**,**d**) TEM (transmission electron microscope), (**e**,**f**) HRTEM (High resolution TEM) spectra, inset (1) of Figure 2f is SAED (selected area electron diffraction) with the 2 1/nm of scale, inset (2) and (3) of Figure 2f the corresponding high-magnification TEM images.

SEM images of NiO/Ni@C-2 composite nanomaterials (Figure 2a,b) show the micro morphology of sample with presence of porous structure and uniformly distributed spherical nanoparticles. The high-magnification SEM image further confirms porous structure of the sample, implying a high specific area. TEM images (Figure 2c–f) also further confirm the micromorphology of porous structure with uniformly distributed spherical nanoparticles. High resolution TEM (HRTEM) images show the spherical nanoparticles in size of approximately 20 nm encapsulated by layered structure. The lattice distances of spherical nanoparticle are approximately 0.25 nm corresponding to the (111) plane of NiO. The selected area electron diffraction (SAED) displays the typical characteristics of multiple rings (inset (1) of Figure 2f). The calculated lattice distances are 0.339, 0.217 and 0.124 nm, which correspond to (002), (100) and (110) crystal surfaces of graphite respectively. These results further confirmed the existence of graphite phase carbon and polycrystalline NiO nanoparticles, indicating the successful synthesis of a few carbon layer coated NiO composite nanomaterials. Further, the lattice distances of the layered structure are 0.34 nm, indicating the presence of graphite phase carbon layer on the surface of NiO nanoparticles. In conclusion, the formation of porous structure can be attributed to two reasons: (1) the gas release in the process of liquid-phase combustion; (2) the formation of etching to remove magnesium oxide and part of nickel oxide nanoparticles. NiO nanoparticles are generated in the original position during combustion and anchored on the surface of the carbon nanosheet, which promote the rapid electron transfer between NiO and the underlying carbon nanosheet.

**Figure 3:**
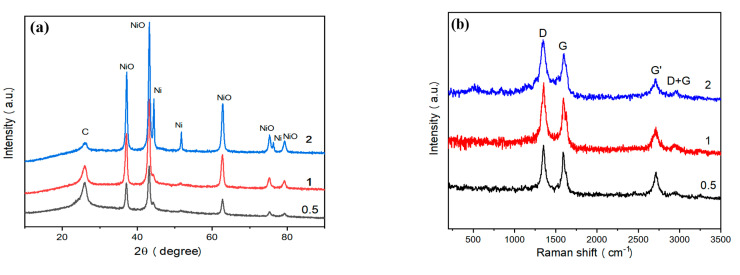

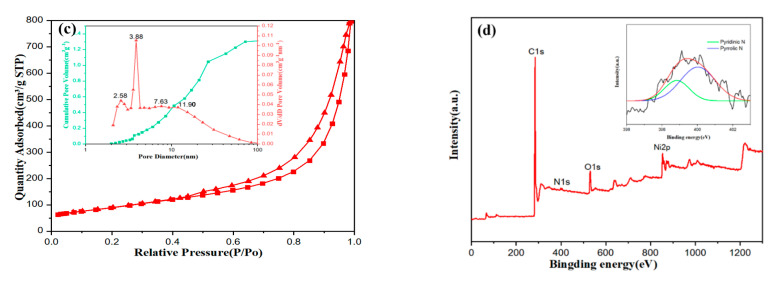
Phase characterization of NiO/Ni@C composite nanomaterials. (**a**) XRD (X-ray diffraction) spectra of the products at different precursors. (**b**) Raman spectra of NiO/Ni@C composite nanomaterials. (**c**) Adsorption and desorption curve of nitrogen. The corresponding pore diameter distribution curve is illustrated in figure c. (**d**) XPS (X-ray photoelectron spectroscopy) spectrum of NiO/Ni@C-2.

The phase composition of recovered samples was analyzed by XRD (Figure 3a). The peaks appearing at 37.3°, 43.4°, 62.9°, 75.5°, and 79.4° are consistent with the characteristic peaks of NiO [26]. The peak appearing at 26.5° is consistent with (002) crystal surface of graphite [25]. The peaks appearing at 44.5°, 51.8° and 76.3° were consistent with the characteristic peaks of Ni [26]. Based on the intensity of the above characteristic peaks, the Ni content is very low in NiO/Ni@C-0.5 and NiO/Ni@C-1. However, NiO/Ni@C-2 sample consists of graphite, NiO, and Ni. Furthermore, the absence of Ni phase in NiO/Ni@C-0.5 and NiO/Ni@C-1 indicate that the Ni content in synthesized samples can be adjusted by the mass of nickel nitrate in the experiment.

Raman spectra (see Figure 3b) show four typical bands of carbon phase, including D band (1350 cm^−1^), G band (1592 cm^−1^), G′ band (2710 cm^−1^), and D + G band (2940 cm^−1^), respectively. D bands reveals the vibration of defects such as disorder and doping in the graphite hexagonal substrate plane. The intensity ratio of D band to G band (I_D_/I_G_) is used to estimate the disorder of graphite phase. Therefore, the intense D band observed in recovered samples and the high ratio of I_D_/I_G_ (1.07) indicate the higher degree of disorder, and a large number of topological defects. The Raman spectrum of NiO/Ni@C-1 sample presents a strong G′ band, which further indicates the good crystallinity of the carbon phase in recovered samples, implying their high conductivity values. Figure 3c presents the BET surface area and DFT pore diameter distribution analysis based on low-temperature nitrogen adsorption measurement. The result indicates that NiO/Ni@C has a type II isotherm with a H3 hysteresis loop according to International Union of Pure and Applied Chemistry (IUPAC) classification [27]. The sample features a specific surface area of 326 m^2^ g^−1^ and a total pore volume of 1.235 cm^3^ g^−1^. The large specific surface area value is mainly due to the molecular level mixing of the reaction precursor and the volume expansion resulting from the gas formation during combustion, such as N_2_, H_2_O (g). The pore size distribution diagram (inset of Figure 3c) indicates that the pore structure of NiO/Ni@C-2 is hierarchical. As shown in the inset of Figure 3c, the mesopores are widely distributed at 1–30 nm, mainly at 2.58, 7.63 and 11.90 nm. Moreover, the peak centered at 3.88 nm is a common artefact due to cavitation. The hierarchical level of porous structure provides abundant reactive sites and enhance the electrochemical performance [25].

The XPS spectrum of NiO/Ni@C-2 (Figure 3d) shows the element of the sample, including C (C1s peak, 284 eV), N (N1 s peak, 400 eV), O (O 1s peak, 532 eV) and Ni (Ni2p-3/2, 853 eV and Ni2p-1/2, 872 eV). The nitrogen content of NiO/Ni@C-2 is 1.94%. The recorded N 1s XPS spectrum of NiO/Ni@C-2 was fitted using Gaussian–Lorentzian peak with non-linear Shirley background correlation. The corresponding peaks were assigned to pyridine (398 eV) and pyridine (399 eV) types of doping [28], which may improve the electrochemical performance of NiO/Ni@C-2 [20,29,30].

### 3.2. Electrochemical Characterization of NiO/Ni@C Composite Nanomaterials

**Figure 4:**
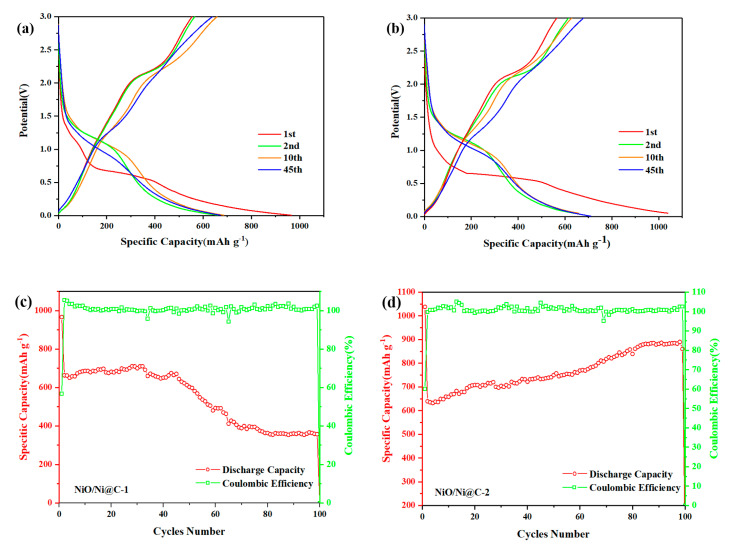
Galvanostatic discharge/charge voltage profiles of NiO/Ni@C-1 (**a**) and NiO/Ni@C-2 (**b**) for the inchoate two cycles, the 10th and 45th cycles between 0.01 and 3.00 V at a current density of 0.1 mA g^−1^. Capacity vs cycle number and the corresponding coulombic efficiency of NiO/Ni@C-1 (**c**) and NiO/Ni@C-2 (**d**) at 0.1 A g^−1^ for 100 cycles.

The working electrodes made from recovered samples were utilized for electrochemical characterization. Figure 4a,b displays the charging/discharging behavior curve of NiO/Ni@C-1 and NiO/Ni@C-2 for the initial two cycles, and 10th and 45th cycles with a voltage range of 0.01–3 at a current density of 0.1 A g^−1^. The first discharge capacity of both samples is 966 mAh g^−1^ and 1038 mAh g^−1^, which are higher than the theoretical one for NiO → Ni reduction. This phenomenon could be attributed to the formation of the solid electrolyte interphase (SEI) coating on the particles during the discharge process [31] and the pseudo-capacitive effect of incorporated nitrogen [20,32]. The initial discharge capacity for the NiO/Ni@C-2 (1038 mAh g^−1^) is higher than that of the NiO/Ni@C-1 (966 mAh g^−1^). It may be attributed to the large quantity of lattice defects in the NiO/Ni@C-2, which was formed owing to that the higher content of nickel nitrate in precursor solution of NiO/Ni@C-2 provides more reaction energy for combustion process as oxidant. These defects may provide sites for the insertion of Li ions [33]. Moreover, Ni nanoparticles can improve the reaction progress of the first charge reaction of NiO anode materials and the conductivity and cycle stability of the materials [34]. However, the irreversible capacity of samples up to 548 mAh g^−1^ and 623 mAh g^−1^ is mainly attributed to the incomplete decomposition of both of the SEI and Li_2_O [35,36]. However, the reversible capacity of both samples maintains a desired value of ca. 650–700 mAh g^−1^ between the 2nd and 45th cycles, which are 1.7 times higher than the theoretical capacity of graphite (372 mAh g^−1^). The first coulombic efficiency of NiO/Ni@C-2 (60.1%) higher than that of NiO@C-1 (56.8%) owing to the catalytic activity induced by highly dispersed Ni nanoparticles in NiO/Ni@C-2 [33]. More SEI will decompose in this the NiO/Ni@C-2 compared to the NiO/Ni@C-1. Beyond that, the decomposition of Li_2_O will be more complete owing to high active Ni content in sample NiO/Ni@C-2 for the reverse reaction (NiO + 2Li ↔ Li_2_O + Ni).

Figure 4c,d display the cycling performance and coulombic efficiency of NiO/Ni@C-1 and NiO/Ni@C-2 at a current density of 0.1 A g^−1^. The results show that the NiO/Ni@C-2 nanocomposite exhibits high performance of cycling. Before the initial 40th cycle, the reversible capacity for the NiO/Ni@C-2 higher than that of NiO@C-1 due to the uniformly distributed Ni nanoparticles in the composite. In addition, the presence of Ni nanoparticles in NiO/Ni@C-2 may improve the electric conductivity [33]. After the 40th cycle, the reversible capacity of NiO/Ni@C-1 decrease to 374 mAh g^−1^ gradually. The capacity loss is due to the formation of irreversible lithium ion traps in SEI membranes and/or lattices, and electrolyte decomposition [32,37]. The NiO/Ni@C-2 exhibits better recycling performance compare to NiO/Ni@C-1, the capacity increases with the cycles number. Furthermore, the reversible capacity for NiO/Ni@C-2 nanocomposite is up to 890 mAh g^−1^ after 100th cycles, and the coulombic efficiency at approximately 100%. The capacity increase of the charge and discharge cycle is due to the presence of NiO and other metal oxide anode, such as CuO, and can be attributed to the catalytic activity of the metal in anode [38]. The activated level hierarchical structure of porous carbon after the initial capacity attenuation may also lead to the increase of capacity [39].

**Figure 5:**
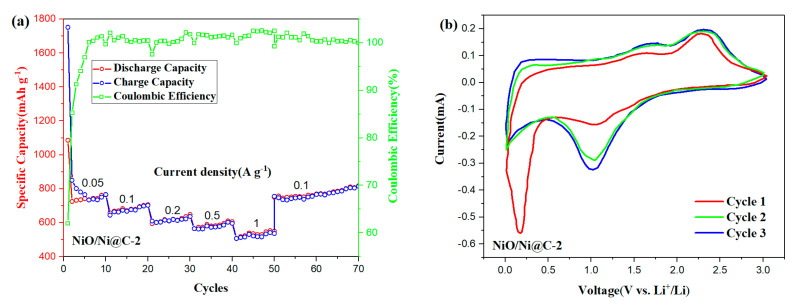
(**a**) Rate capacity at different specific currents, and (**b**) CV (Cyclic voltammograms) curve of the initial cycle of NiO/Ni@C-2 composites.

Figure 5a shows that NiO/Ni@C-2 exhibits a desirable rate capability and cycling stability from 0.05 to 1 A g^−1^ as LIB anode. The reversible capacities of 745, 671, 620, 580, and 530 mAh g^−1^ were obtained at the current densities of 0.05, 0.1, 0.2, 0.5, and 1 A g^−1^, respectively. Beyond that, the discharge specific capacity rises to 750 mAh g^−1^ after the current density decreases back to 0.1 A g^−1^, indicating that the NiO/N@C composite anode had a high cycling stability and good reversibility. Cyclic voltammograms (CV) are further studied in detail to track the Li-ion storage process. As shown in Figure 5b, an apparent reduction peak centered at 0.24 V is observed in the first discharge of NiO/Ni@C-2, which behavior could be attributed to irreversible reactions leading to the transformation of NiO into Ni with amorphous Li_2_O generation (NiO + 2Li^+^ + 2e^−^ → Ni + Li_2_O), and the formation of SEI film. The reduction peaks at 1.15 V indicate the lithium ion dissociation from the pore structure or defect. A strong oxidation peak appeared at 2.26 V, corresponding to oxidation during charging (Ni + Li_2_O → NiO + 2Li^+^ + 2e^−^), and Li binding with heteroatoms on the anode surface [35,40]. After the first cycle, the CV curves approximately overlapped, indicating that the NiO/ Ni@C-2 composite nanomaterials have good reversibility and cycle stability as anode materials for LIBs.

## 4. Conclusions

In conclusion, nitrogen-doped carbon-encapsulated NiO/Ni nanocomposite materials were successfully synthesized by SCS using carbon dioxide as the carbon source. Furthermore, through this method, Ni nanoparticle content can be adjusted by the mass content of nickel nitrate in the reaction solution. As the anode of the lithium ion battery, the nanocomposite material exhibits a high capacity and excellent circulation performance. The composite with abundant Ni nanoparticles (NiO/Ni@C-2) exhibits a higher initial discharge capacity (1038 mAh g^−1^), higher initial coulombic efficiency (60.1%), and better cycling performance compared to the NiO/Ni@C-1. The reversible capacity of lithium ion battery with NiO/Ni@C-2 electrode is 890 mAh g^−1^ after 100 cycles with a current density of 0.1 A g^−1^, higher than the theoretical value of pure NiO (718 mAh g^−1^). The excellent electrochemical performance for NiO/Ni@C-2 composite is mainly attributed to the following reasons. (1) The porous structure and high specific surface area of the carbon matrix can buffer the volume expansion in the process of charge and discharge cycle and the NiO/Ni@C exposed to the electrolyte increases the contact area with the electrolyte. (2) The size effect of nanoparticles further buffers the change of volume and structural stress because of the easier path of ion insertion and removal. (3) The active Ni nanoparticles promote the decomposition of Li_2_O and SEI, leading to the improvement of the conductivity of NiO/Ni@C samples. (4) The nitrogen doping of NiO/Ni@C provides abundant active reaction sites and further improves the electrochemical performance.

## References

[1] Zou Y., Wang Y. (2011). NiO nanosheets grown on graphene nanosheets as superior anode materials for Li-ion batteries. Nanoscale.

[2] Paek S.-M., Yoo E., Honma I. (2009). Enhanced cyclic performance and lithium storage capacity of SnO_2_/graphene nanoporous electrodes with three-dimensionally delaminated flexible structure. Nano Lett..

[3] Poizot P., Laruelle S., Grugeon S., Dupont L., Tarascon J.-M. (2000). Nano-sized transition-metal oxides as negative-electrode materials for lithium-ion batteries. Natural.

[4] Zhao Y., Ma C., Li Y., Chen H., Shao Z. (2015). Self-adhesive Co_3_O_4_/expanded graphite paper as high-performance flexible anode for Li-ion batteries. Carbon.

[5] Singh A.K., Sarkar D., Khan G.G., Mandal K. (2014). Hydrogenated NiO nanoblock architecture for high performance pseudocapacitor. ACS Appl. Mater. Interfaces.

[6] Wei C., Pang H., Zhang B., Lu Q., Liang S., Gao F. (2013). Two-dimensional β-MnO_2_ nanowire network with enhanced electrochemical capacitance. Sci. Rep..

[7] Wen X., Zhang W., Yang S. (2003). Synthesis of Cu(OH)_2_ and CuO nanoribbon arrays on a copper surface. Langmuir.

[8] Liu Y., Li J., Zhang Q., Zhou N., Uchaker E., Cao G. (2011). Porous nanostructured V_2_O_5_ film electrode with excellent Li-ion intercalation properties. Electrochem. Commun..

[9] Nuli Y., Zhang P., Guo Z.P., David W., Liu H.K., Yang J., Wang J.L. (2009). Nanostructured NiO/C composite for lithium-ion battery anode. J. Nanosci. Nanotechnol..

[10] Jiapan Z., Mi H., Jide W., Qingxia C., Jialiang Z. (2015). Preparation and lithium storage performance of NiO/ C@CNT anode material. Rare Met. Mater. Eng..

[11] Cheng M.-Y., Pan C.-J., Hwang B.J. (2009). Highly-dispersed and thermally-stable NiO nanoparticles exclusively confined in SBA-15: Blockage-free nanochannels. J. Mater. Chem..

[12] Fan Y., Ma Z., Wang L., Dong Y., Jiang T., Li Z., Liu L., Shao G. (2018). In-situ synthesis of NiO foamed sheets on Ni foam as efficient cathode of battery-type supercapacitor. Electrochim. Acta.

[13] Kim J.Y., Lee S.-H., Yan Y., Oh J., Zhu K. (2012). Controlled synthesis of aligned Ni-NiO core-shell nanowire arrays on glass substrates as a new supercapacitor electrode. RSC Adv..

[14] Liu L., Guo Y., Wang Y., Yang X., Wang S., Guo H. (2013). Hollow NiO nanotubes synthesized by bio-templates as the high performance anode materials of lithium-ion batteries. Electrochimica Acta.

[15] Wu J., Yin W.-J., Liu L.-M., Guo P., Liu G., Liu X., Geng D., Lau W.-M., Liu H., Liu L.-M. (2016). High performance NiO nanosheets anchored on three-dimensional nitrogen-doped carbon nanotubes as a binder-free anode for lithium ion batteries. J. Mater. Chem. A.

[16] Li X., Chen Y., Zhou L., Mai Y.-W., Huang H. (2014). Exceptional electrochemical performance of porous TiO2–carbon nanofibers for lithium ion battery anodes. J. Mater. Chem. A.

[17] Zhao B., Song J., Liu P., Xu W., Fang T., Jiao Z., Zhang H., Jiang Y. (2011). Monolayer graphene/NiO nanosheets with two-dimension structure for supercapacitors. J. Mater. Chem..

[18] Kim N.D., Kim W., Joo J.B., Oh S., Kim P., Kim Y., Yi J. (2008). Electrochemical capacitor performance of N-doped mesoporous carbons prepared by ammoxidation. J. Power Sources.

[19] Chen M. (2018). Microporous N-doped carbon electrochemical catalyst derived from polyacrylamide hydrogel for oxygen reduction reaction in alkaline media. Int. J. Electrochem. Sci..

[20] Wang X., Li Q., Zhang Y., Yang Y., Cao Z., Xiong S. (2018). Synthesis and capacitance properties of N-doped porous carbon/NiO nanosheet composites using coal-based polyaniline as carbon and nitrogen source. Appl. Surf. Sci..

[21] Lai H., Wu Q., Zhao J., Shang L., Li H., Che R., Lyu Z., Xiong J., Yang L., Wang X. (2016). Mesostructured NiO/Ni composites for high-performance electrochemical energy storage. Energy Environ. Sci..

[22] Patil K.C., Hegde M.S., Rattan T., Aruna S.T. (2008). Zirconia and related oxide materials. Chemistry of Nanocrystalline Oxide Materials: Combustion Synthesis, Properties and Applications.

[23] Li F.-T., Ran J., Jaroniec M., Qiao S. (2015). Solution combustion synthesis of metal oxide nanomaterials for energy storage and conversion. Nanoscale.

[24] Varma A., Mukasyan A.S., Rogachev A.S., Manukyan K.V. (2016). Solution combustion synthesis of nanoscale materials. Chem. Rev..

[25] Xu C., Chen P., Liu K., Gao X., Du L. (2019). CO_2_ conversion into N-doped carbon nanomesh sheets. ACS Appl. Nano Mater..

[26] Wang L., Jiao Y., Yao S., Li P., Wang R., Chen G. (2019). MOF-derived NiO/Ni architecture encapsulated into N-doped carbon nanotubes for advanced asymmetric supercapacitors. Inorg. Chem. Front..

[27] Thommes M., Kaneko K., Neimark A.V., Olivier J.P., Rodriguez-Reinoso F., Rouquerol J., Sing K.S. (2015). Physisorption of gases, with special reference to the evaluation of surface area and pore size distribution (IUPAC Technical Report). Pure Appl. Chem..

[28] Xu Z., Zhuang X., Yang C., Cao J., Yao Z., Tang Y., Jiang J., Wu D., Feng X. (2016). Nitrogen-doped porous carbon superstructures derived from hierarchical assembly of polyimide nanosheets. Adv. Mater..

[29] Wang B., Li Z., Zhang J., Xia Z., Yang H., Fan M., Yu Y. (2019). N-doped 3D interconnected carbon bubbles as anode materials for lithium-ion and sodium-ion storage with excellent performance. J. Nanosci. Nanotechnol..

[30] Liang J., Yuan C., Li H., Fan K., Wei Z., Sun H., Ma J. (2017). Growth of SnO_2_ nanoflowers on N-doped carbon nanofibers as anode for Li- and Na-ion batteries. Nano-Micro Lett..

[31] Debart A., Revel B., Dupont L., Montagne L., Touboul M., Leriche J.-B., Tarascon J.-M. (2003). Study of the reactivity mechanism of M_3_B_2_O_6_ (with M: Co, Ni, and Cu) toward lithium. Chem. Mater..

[32] Adams R.A., Syu J.-M., Zhao Y., Lo C.-T., Varma A., Pol V.G. (2017). Binder-free N- and O-rich carbon nanofiber anodes for long cycle life K-ion batteries. ACS Appl. Mater. Interfaces.

[33] Huang X., Tu J., Zhang B., Zhang C., Li Y., Yuan Y., Wu H. (2006). Electrochemical properties of NiO–Ni nanocomposite as anode material for lithium ion batteries. J. Power Sources.

[34] Ni S., Li T., Lv X., Yang X., Zhang L. (2013). Designed constitution of NiO/Ni nanostructured electrode for high performance lithium ion battery. Electrochim. Acta.

[35] Ni S., Lv X., Ma J., Yang X., Zhang L. (2014). A novel electrochemical reconstruction in nickel oxide nanowalls on Ni foam and the fine electrochemical performance as anode for lithium ion batteries. J. Power Sour..

[36] Gong Y., Zhang M., Cao G. (2015). Chemically anchored NiO_x_–carbon composite fibers for Li-ion batteries with long cycle-life and enhanced capacity. RSC Adv..

[37] Tang J., Lugo C.E.Z., Guzmán S.F.A., Daniel G., Kessler V.G., Seisenbaeva G.A., Pol V.G. (2016). Pushing the theoretical capacity limits of iron oxide anodes: Capacity rise of γ-Fe2O3nanoparticles in lithium-ion batteries. J. Mater. Chem. A.

[38] Débart A., Dupont L., Poizot P., Leriche J.-B., Tarascon J.M. (2001). A Transmission electron microscopy study of the reactivity mechanism of tailor-made CuO particles toward lithium. J. Electrochem. Soc..

[39] Zhang C., Huang B., Miao X., Feng Z., Huang Y. (2018). An environmental benign approach to high performance anode for Li-ion battery: N-rich porous carbon from Cr(VI)-polluted water treatment. Mater. Lett..

[40] Xu C., Chen S., Du L., Li C., Gao X., Liu J., Qu L., Chen P. (2018). Scalable conversion of CO_2_ to N-doped carbon foam for efficient oxygen reduction reaction and lithium storage. ACS Sustain. Chem. Eng..

